# The association between psychological stress and miscarriage: A systematic review and meta-analysis

**DOI:** 10.1038/s41598-017-01792-3

**Published:** 2017-05-11

**Authors:** Fan Qu, Yan Wu, Yu-Hang Zhu, John Barry, Tao Ding, Gianluca Baio, Ruth Muscat, Brenda K. Todd, Fang-Fang Wang, Paul J Hardiman

**Affiliations:** 1 Women’s Hospital, School of Medicine, Zhejiang University, No.1 Xueshi Road, Hangzhou, 310006 Zhejiang P. R. China; 2 Institute of Women’s Health, University College London, Rowland Hill Street, London, NW3 2PF UK; 3 Department of Statistical Science, University College London, Gower Street, London, WC1E 6BT UK; 4 Library Services, University College London, Gower Street, London, WC1E 6BT UK; 5 Psychology Department, School of Social Sciences, City University London, Northampton Square, London, EC1V 0HB UK; 6 EBSCO Health for UK North & Ireland at EBSCO Information Services, 4th Floor Kingmaker House, Station Road, New Barnet, EN5 1NZ UK

## Abstract

This systematic review and meta-analysis was designed to investigate whether maternal psychological stress and recent life events are associated with an increased risk of miscarriage. A literature search was conducted to identify studies reporting miscarriage in women with and without history of exposure to psychological stress (the only exposure considered). The search produced 1978 studies; 8 studies were suitable for analysis. A meta-analysis was performed using a random-effects model with effect sizes weighted by the sampling variance. The risk of miscarriage was significantly higher in women with a history of exposure to psychological stress (OR 1.42, 95% CI 1.19–1.70). These findings remained after controlling for study type (cohort and nested case-control study OR 1.33 95% CI 1.14–1.54), exposure types (work stress OR 1.27, 95% CI 1.10–1.47), types of controls included (live birth OR 2.82 95% CI: 1.64–4.86). We found no evidence that publication bias or study heterogeneity significantly influenced the results. Our finding provides the most robust evidence to date, that prior psychological stress is harmful to women in early pregnancy.

## Introduction

Spontaneous pregnancy loss is the most common complication of pregnancy^1, 2^; it occurs before 24 weeks of gestation in around 20% of pregnancies^3–5^ and in 12–15% of clinically recognized pregnancies^6^. However, many cases of miscarriage are unreported; especially those involving early fetal loss, so the incidence may be even higher^7^. Only a small proportion (<10%) of women who experience miscarriage report recurrent pregnancy loss^7^ and as many as a third of pregnancy losses are not linked to chromosomal abnormalities^5^. Miscarriage is often associated with high levels of distress for women, their partners and families; therefore, every potential cause of miscarriage should be investigated. The evidence relating stress to spontaneous miscarriage is conflicting. Women reporting one or more recent negative life events prior to miscarriage were twice as likely to have a chromosomally normal spontaneous abortion^8^, even after adjusting for life-style factors^9^ and a similar two-fold increase in miscarriage was found in women with a history of exposure to psychological stress^1^. Stress (e.g. financial or marital problems, death, divorce, physical and nonphysical abuse inflicted on a woman by her partner and loss of social support) was also associated with the likelihood of miscarriage among women reporting to an emergency department or admission to hospital^10, 11^.Psychological challenges can include the experience of emotional trauma, social problems, concerns about money, marital/partnership disharmony, work pressure, significant change in personal circumstances as well as prior pregnancy loss^12^. In addition, retrospective studies link increase in workplace demands with adverse pregnancy outcomes including miscarriage^7, 13^.

On the other hand, possibly because of a desire not to exacerbate women’s concerns, many doctors discount any association between stress and miscarriage. In the UK for example, an NHS website (http://www.nhs.uk/Conditions/Miscarriage/Pages/Causes.aspx) advises mothers that the risk of miscarriage being related to a mother’s emotional state is a “common misconception”. Perhaps because of a lack of evidence, opposing opinions regarding stress as a cause of miscarriage are widely held.

Further evidence of a link between stress and adverse reproductive outcomes comes from animal studies; for example, among non-domesticated animals, re-location and exposure to unfamiliar conspecifics can lead to sufficient stress to cause miscarriage^14^. The negative effect of stress on the nervous, endocrine and immune systems of mice is also associated with abortion^15^.

The belief that stress at the time of conception or during pregnancy can harm their baby, causing problems such as miscarriage, is widely held amongst women. For example, 76% of women attending an antenatal clinic in the USA, thought that a mother’s stress can negatively affect pregnancy outcome, with 35% believing that pregnant women should avoid upsetting things like violent programs or funerals^16^. Women in that study were interpreting the term “stress” in its psychological form i.e. they experience negative emotionality when their physical or psychological well-being is threatened. Some doctors and midwives share this view although they know that fetal chromosomal abnormality is present in around two thirds of cases of early pregnancy failure^5^. Other risk factors for miscarriage include increased maternal age, obesity, caffeine^17^, alcohol^7, 18, 19^, cigarette smoke^20, 21^ and exercise^22^.

The diverse views held by women and healthcare professionals demonstrate the need for evidence in this vital area of human wellbeing. Awareness of the effects of psychosocial stress could lead to improved strategies for screening psychological support and changes in employment practices. This systematic review and meta-analysis as therefore designed to investigate whether maternal psychological stress is associated with an increased risk of miscarriage.

## Results

### Characteristics of included studies

**Figure 1:**
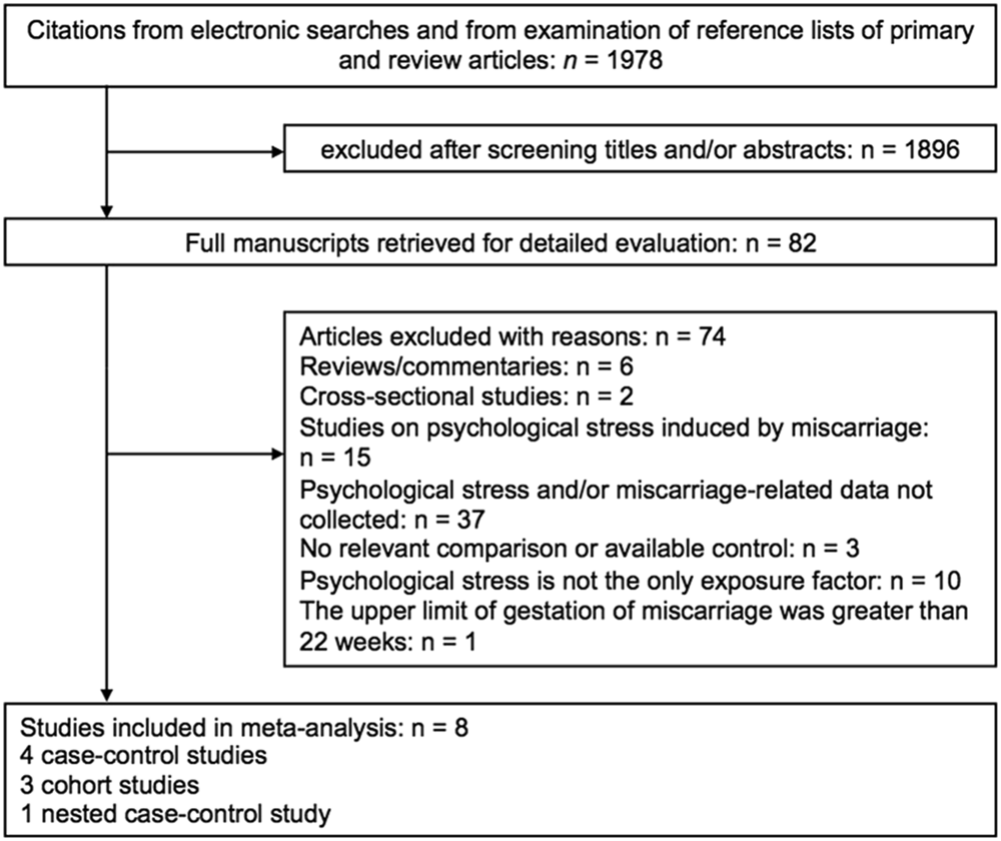
Flow diagram illustrating the selection procedure of relevant articles reporting on the association between psychological stress and miscarriage.

The search strategy produced 1978 studies; of which, 1896 studies with irrelevant title and/or abstract were excluded. Full text papers were retrieved for 82 studies and 74 studies were further excluded in compliance with the criteria defined in methods section. A final number of 8 studies were included for the meta-analysis. Details of the study selection process were presented in Fig. 1.

Characteristics of all studies included in the systematic review were shown as Table 1. Of the 8 included studies, 4 are case-control studies, 3 are cohort studies, and 1 is a nested case-control study. The sample size in these studies ranged from 96 to 6945. All of the 8 included studies reported odds ratio (OR) with 95% confidence interval (CI) as the outcome measure of the association between psychological stress and miscarriage^1, 2, 7, 8, 10, 11, 13, 23^.

**Table 1:** Characteristics of all studies included in the systematic review.

First author, year published	Country	Year	Design	Total sample size	Sample size related to our meta-analysis	Exposures	Reference group	Stress measurement	Effect estimates	Matched or adjusted confounders	NOS score
Bashour H^1^	Syria	1999	Case-control	1098	1098	Psychological stress	Women delivered normal babies at term	Questionnaires (the cases and controls were interviewed by trained midwives, using a structured questionnaire).	OR	No	6
Boyles SH^8^	USA	1995–1997	Nested case-control	970	970	Life events	Women maintained their pregnancy	A modified life event inventory (the participants were questioned about different categories of major life events, including death,debt burden, legal problems, and personal relationships).	OR	Tobacco use, cocaine use, alcohol use, prenatal care, living with the father	9
Brandt L, 1992^12^	Denmark	1983–1985	Cohort	6945	4500	Work stress	ND	A questionnaire about stress-related job characteristics (a questionnaire about the information on occupational status, job title, stress-related job characteristics, ergonomic work load, exposure to organic solvents, exposure to video display terminals, lifestyle factors, and health factors during pregnancy).	OR	Previous pregnancies	6
Fenster L, 1995^22^	USA	1990–1991	Cohort	3953	3953	Work stress	Women maintained their pregnancy	An abbreviated version of instrument (the instrument is based on the concepts that job stress results from high psychological demands in combination with low control over those demands and that social support at work can ameliorate the effects of stressful work).	OR	Maternal age, race, pregnancy history, marital status, alcohol, cigarette, and caffeine consumption	9
Maconochie N^7^	UK	2001	Case-control	6719	5272	Work stress	Women maintained their pregnancy	Questionnaires (Stage1:a short “screening” questionnaire; Stage2:a more lengthy questionnaire; Stage3:a shortened version of the stage2 questionnaire, containing only the questions relating to biological, socio-demographic and behavioral details of last pregnancy which in relation to the most recent miscarriage).	OR	Year of conception, maternal age, previous miscarriage and previous live birth, nausea	7
Meaney S^2^	Ireland	2012	Cohort	417	417	Psychological stress	Women maintained their pregnancy	Questionnaires and psychometric tests (detailed lifestyle questionnaires, including common risk factors for miscarriage, and psychometric tests, including the 36-Item Health Survey, the Maternity Social Support Scale, the Revised Life Orientation Test and the Perceived Stress Scale).	OR	ND (without detailed description of the adjusted confounders)	7
Nelson DB^10^	USA	1999–2000	Case-control	326	326	Psychological stress	Women maintained their pregnancy	Perceived Stress Scale; Prenatal Social Environment Inventory; Index of Spousal Abuse.	OR	Maternal age, gestational age, cigarette and cocaine use, prior spontaneous abortion	8
O’Hare T^11^	UK	ND	Case-control	96	96	Life events	Women giving birth in hospital	Life Events and Difficulties Schedule (the women were interviewed in hospital).	OR	Age, marital status, social class distribution, woman’s or partner’s employment status, numbers of children or adults in household, obstetric history	8

Note: NOS: Newcastle-Ottawa Scale; OR: Odds Ratio; CI: confidence interval; ND: Not Described; BMI: Body Mass Index.

### Quality assessment and publication bias

Results from Newcastle-Ottawa Scale (NOS) indicated that six of our included studies were rated7–9 stars whereas two studies were rated 6 stars (shown as Supplementary Table S1). Seven studies achieved high comparability by adjusting for or matching on at least one of the following confounders: maternal age, gestational age, pregnancy history, caffeine and tobacco consumption, and social support. Begg’s rank correlation test (p = 0.536), Egger’s linear regression test (p = 0.170) and the funnel plot (shown as Supplementary Figure S1) suggested that there was no significant publication bias.

### Effect size analysis

**Figure 2:**
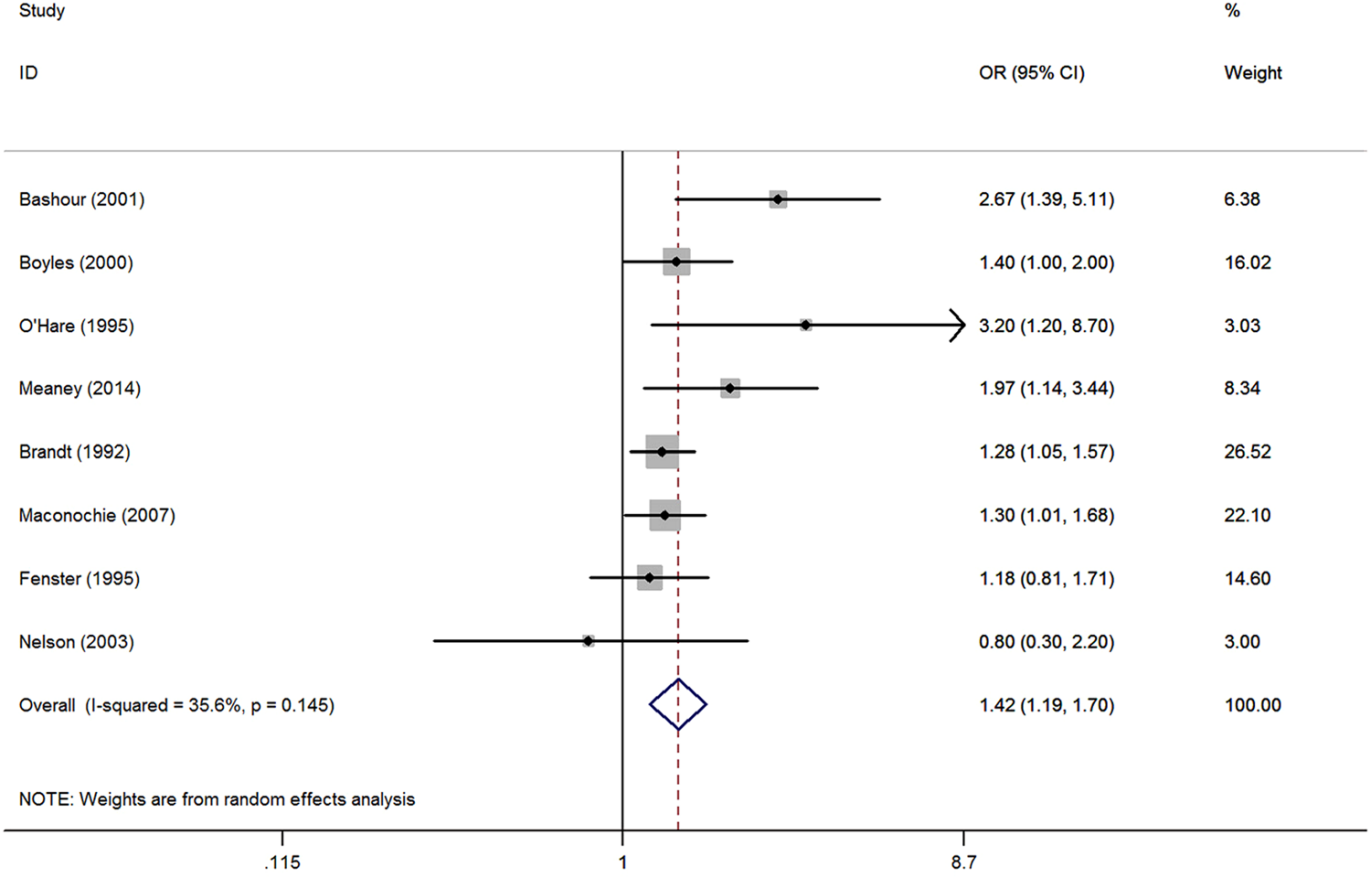
Meta-analysis of eight studies about the effect of maternal psychological stress on miscarriage. (note: OR, odds ratio; CI, confidence interval).

We evaluated for the outliers before starting the analysis, and found no extreme values. As presented in Fig. 2, the overall pooled OR was 1.42 (95% CI 1.19 to 1.70) with moderate heterogeneity (I^2^ = 35.6%), indicating that maternal psychological stress is significantly associated with an increased risk of miscarriage.

Subgroup analysis was conducted to explore the sources of the heterogeneity (presented in Table 2). There was positive association between psychological stress and miscarriage for the type of study (OR: 1.69 for case-control studies; 1.33 for cohort and nested case-control study).However, the OR was statistically significant only for cohort and nested case-control studies (OR, 1.33; 95% CI, 1.14–1.54; P < 0.001). Moreover, substantial heterogeneity (I^2^ = 62.1%) was reported for case-control studies, whereas for cohort and nested case-control study, heterogeneity was low (I^2^ = 0.0%).

**Table 2:** Results of subgroup analyses.

Factor	Number of studies	OR (95% CI)	P Value	I^2^(%), p Value*
Study type				
Case-control	4	1.69 (0.99 to 2.88)	0.054 < 0.001	62.1, 0.048
Cohort + Nested case-control	4	1.33 (1.14 to 1.54)	<0.001	0.0, 0.464
Exposures				
Psychological stress	3	1.80 (1.01 to 3.19)	0.045	49.5, 0.138
Life events	2	1.85 (0.86 to 3.97)	0.116	58.1, 0.123
Work stress	3	1.27 (1.10 to 1.47)	0.001	0.0, 0.911
Control (Miscarriage vs)				
Live birth	2	2.82 (1.64 to 4.86)	<0.001	0.0, 0.765
Ongoing pregnancy	5	1.33 (1.12 to 1.57)	0.001	0.0, 0.485
Undefined	1	1.28 (1.05 to 1.57)	0.016	—
Quality of studies				
Low	2	1.73 (0.85 to 3.51)	0.130	77.6, 0.034
High	6	1.38 (1.13 to 1.70)	0.002	21.7, 0.270
With/without adjusted confounders				
With	7	1.34 (1.16 to 1.54)	<0.001	9.2, 0.358
Without	1	2.67 (1.39 to 5.12)	0.003	—

Note: OR: Odds Ratio; CI: confidence interval; *p Value for heterogeneity.

Exposures in the included studies were divided into three types: psychological stress, life events and work stress. In terms of subgroup analysis based on exposure factors, we found that there was no heterogeneity (I^2^ = 0.0%) between studies concerning work stress^7, 13, 23^. We found that work stress was significantly associated with an increased risk of miscarriage (OR, 1.27; 95% CI, 1.10–1.47; P = 0.001).

In subgroup analysis according to the types of controls, psychological stress was observed to have the greatest impact on miscarriage when the comparison group consisted of women who had a live birth (OR, 2.82; 95% CI, 1.64–4.86; P < 0.001)^1, 11^.

We further categorized the eight included studies by NOS scores; heterogeneity decreased (I^2^ = 21.7%) when the two lower quality studies were excluded^1, 13^. After removing the study not controlling for potential confounding factors^1^, the pooled OR slightly decreased to 1.34 (95% CI 1.16 to 1.54; I^2^ = 9.2%). Results from sensitivity analysis (i.e. excluding one study at a time) demonstrated that none of the studies caused significant heterogeneity compared with the rest, or strongly influenced the results (shown as Supplementary Figure S2).

## Discussion

The results of this meta-analysis support the belief that psychological stress before and during pregnancy is associated with miscarriage. A view held by some medical practitioners and around three quarters of pregnant women, but most often dismissed by doctors and other health care professionals. Whilst chromosomal abnormalities underlie many cases of early pregnancy loss, the present results show that these psychological factors can increase the risk by approximately 42%.

Psychological stress can influence well-being through associated health-impairing behaviors and through physiological responses which affect vascular, immune, metabolic or neuroendocrine functions^24^. The experience of stress can originate in a wide range of circumstances and is defined as “any situation that overwhelms our ability to cope”^25^. Therefore, the experience of stress varies, not only by an individual’s internal resources but also by the social and material support which is available to them. Effects are difficult to assess as physiological responses to stress vary with its intensity and duration, and are contingent on the genetic vulnerability and life history of the affected individual^26^. For example, the degree of stress experienced in infancy and childhood have implications for the individual’s subsequent physiological response to stress^25^. Persistent stressors, which are perceived as uncontrollable, are the most damaging to physical and mental health^25^. However, as far as the authors are aware, different classes of stress do not systematically elicit differential physiological responses.

Because of the complexity of the mechanisms and the degree of individual variation in the response to stressors, accurate measurement and comparisons of the experience of stress between individuals or groups of individuals is challenging. Psychological stress is also likely to co-occur with other psychological factors such as anxiety and depression^27^ and may be chronic, acute or transient. A variety of relevant scales have been applied in specific studies, for example, the Perceived Stress Scale^28^, the Pre-natal Social Environment Inventory^29^ and Index of Spousal Abuse^30^ and others pertaining more generally to life events and stress symptoms, thus making simple comparisons between studies difficult. Retrospective reports, both from focal groups and controls, may be especially vulnerable to recall bias. Even a small indicator of prospective miscarriage, or history of previous miscarriage, is likely to produce stress, therefore confounding the direction of effects. In addition, many miscarriages are managed at home and never reported^7^. Estimating miscarriage risk is further complicated by the difficulty in distinguishing the effect of stress from the effects of substances like alcohol, tobacco and caffeine which are taken to relieve stress.

Based on the considerations described above, interpretation of the findings from this meta-analysis is subject to some caution; the included studies also vary by the types of stress under focus and their prospective or retrospective design. The measures of stress vary between studies and do not always assess symptoms directly^8, 13^ and some scales do not specify cut-off points between high and low stress (e.g. Perceived Stress Scale). Participant self-reports are often retrospective with an associated risk of recall bias, as authors generally acknowledge^11, 13^. Whilst the NOS assessment provided a means to assess non-randomized studies, the scoring system itself is not without its drawbacks and criticisms^31^. Study quality is also variable: some offer limited detail on assessment^1, 7, 23^, on case selection procedures^1^ and on timing of assessment in relation to outcome^1^. Therefore we propose the need for high quality research into an association between the experience of stress in a variety of contexts and miscarriage risk.

In the present meta-analysis, on sub analysis, six studies with higher quality showed a significantly increased miscarriage risk in women suffering from psychological stress, but this was not found in the two studies with lower NOS scores. An increased miscarriage risk was found on analysis of cohort and nested case-control but not in case-control studies. For case-controls, the variability is also much larger (leading to an interval including 0). The explanation for this possibly relates to the smaller number of studies (hence larger variability), or perhaps the increased level of heterogeneity intrinsic in case-controls. However inclusion or exclusion of confounders did not affect the results.

The association between psychological stress and miscarriage could result, at least in part, from activation of the hypothalamic-pituitary-adrenal axis by recruitment of hypothalamic neurones which secrete corticotrophin-releasing hormone, increasing pituitary secretion of adrenocorticotrophic hormone secretion and hence of adrenal cortisol^32^. This hormone has direct effects on decidual and placental metabolism but also interacts with progesterone signalling^32^. Stress-related early pregnancy failure could also result from suppression of the hypothalamic-pituitary-gonadal axis^32–35^. Although generally considered a “stress hormone”, prolactin production is decreased by stress in early pregnancy^14, 32, 36^. Since prolactin stimulates progesterone secretion, the reduced levels will decrease progesterone synthesis^37, 38^. Stress also inhibits pituitary human chorionic gonadotropin secretion compounding the effect of prolactin on progesterone release from the corpus luteum^32, 39, 40^. These mechanisms are relevant because progesterone activity is crucial for the maintenance of pregnancy; low levels in early of gestation predicting miscarriage^32^. Among its multiple effects, this hormone contributes to the suppression of maternal immune response to the conceptus^32^.

In summary, the result of this systematic review and meta-analysis support the belief that psychological stress, including life events and occupational stress, in pregnancy is associated with an increased risk of miscarriage and indicates a critical need for further high quality research into the relationship between miscarriage and stress experienced prior to pregnancy and in the early gestational period. Taken together with the serious morbidities already known to be associated with stress (pregnancy induced hypertension, preterm birth and low birth weight), this finding also highlights the need to include a structured psychological assessment in early pregnancy into routine antenatal care. This demonstration that stress contributes to early pregnancy failure could provide the basis for novel and effective interventions in this field. As far as we are aware there have been no randomized trials of psychological therapy to prevent miscarriage, however Liddell, Pattison and Zanderigo^41^ reported a live birth rate of 86% in women with recurrent (≥3) miscarriages who were enrolled into a program of emotional support, compared to 33% in similar women who had no formal supportive care. Twenty-five years later, the results of our meta-analysis, highlight the potential to identify and treat psychological factors which contribute to adverse pregnancy outcomes in the human.

## Methods

### Literature search

We searched the following databases for published articles and conference abstracts and proceedings in consultation with a search methodologist, using the Medline search strategy below (with minor modifications to account for different controlled vocabularies and syntax): MEDLINE (Ovid, 1946 – June, 2016), EMBASE (Ovid, 1980 – June, 2016), PsycINFO (Ovid, 1806 – June, 2016), CINAHL plus (EBSCO, 1937 – June, 2016), Maternity & Infant Care (Ovid, 1971 – June, 2016), and Conference Proceedings Citation Index - Science (CPCI-S, 1990 – June, 2016). No language or date restrictions were applied.

Search strategy: (1) exp abortion, spontaneous/; (2) ((tubal or threatened or missed or spontaneous or recurrent or incomplete or inevitable or habitual or septic) adj abortion*).mp;(3) miscarr*.ti,ab;(4) ((pregnancy or embryo) adj3 (loss or failure)).mp; (5) blighted ovum.mp;(6)misbirth.mp; (7) exp fetal death/; (8) ((fetal or foetal or fetus or foetus or intrauterine or antepartum or prenatal) adj (death or resorption or mummification)).ti,ab; (9)or/1–8; (10) exp stress, psychological/;(11)anxiety/; (12)exp stress disorders, traumatic/; (13) panic disorder/; (14)((stress* or distress* or anxiety or PTSD or panic) not oxidative).ti,ab;(15)life change events/; (16) (life adj2 event*).ti,ab;(17)or/10–16; (18) exp risk factors/or exp risk/; (19) risk*.ti,ab;(20) 18 or 19; (21) 9 and 17 and 20.

We only included case-control, cohort (retrospective or prospective) and nested case-control studies for this review and for studies that reported similar or overlapping data, only the latest or those with a larger sample size were considered.

Only studies that included women who had miscarriage (cases) and women with ongoing pregnancy or live birth (controls) were considered for eligibility. Miscarriage or pregnancy loss occurring before the first 22 weeks gestation is defined as the natural death of an embryo or fetus before it is able to survive independently. The following exclusion criteria were applied: (i) psychological stress was induced by miscarriage; (ii) psychological stress and/or miscarriage-related data could not be retrieved; (iii) women had mental or psychological disorders before pregnancy; (iv) no relevant comparison or available control were present; (v) psychological stress before miscarriage (including anxiety, depression, life event, work/job stress, etc.) was not the only exposure factor.

Three authors(F.Q., Y.W., and Y.Z) independently reviewed and selected the articles in compliance with the inclusion/exclusion criteria. Disagreement was resolved by consensus or arbitration.

### Study coding

The following information was recorded or coded for each article: country, year, study design, study population characteristics, total sample size, stress measurement methods of the studies, sample size related to our meta-analysis and outcome data. All the reviewers cross-checked the extracted data repeatedly and any disagreements were resolved by consensus. Authors were contacted for further details if necessary.

The quality of the included studies was assessed independently by three authors (F.Q., Y.W. and Y.Z.) using NOS^42^. Different assessment items were applied to case-control and cohort studies, respectively. For each type of study, eight criteria were used in the assessment, namely (1) for cohort studies: representativeness of exposed cohort, selection of non-exposed cohort, ascertainment of exposure, outcome not present at baseline, comparability of cohorts, assessment of outcome, sufficient follow-up duration, and adequate follow-up; (2) for case-control studies: adequate definition of cases, representativeness of cases, selection of controls, definition of controls, comparability of cases and controls, ascertainment of exposure, same method of ascertainment for cases and controls, and non-response rate. The total score for each study was obtained by summing up stars from each item. More than six stars indicate good quality, whereas 5–6 stars indicate acceptable quality. Disagreements were resolved by consensus.

### Meta-analysis procedures

Data were all presented as OR with 95% confidence interval CI. We firstly evaluated for the outliers, defining as that the individual ORs were more than 2 standard deviations from the mean of all the effect sizes, to see if replacement of extreme values is necessary. The statistical analysis was performed in STATA 12.0 software (StataCorp, College Station, USA). This provided effect sizes weighted by the sampling variance, with a 95% confidence interval and a measure of heterogeneity. DerSimonian and Laird random-effects model was employed, as our effect sizes were assumed to be sampled from a large number of possible sample sizes. Heterogeneity between studies was evaluated using homogeneity statistic (Q), which follows a Chi-square distribution with a degree of freedom of (n-1) where n is the total number of studies included^43^. I^2^ statistic was also used to evaluate the heterogeneity, and results were deemed significance in correspondence of a Chi-squared test with p < 0.10 or I^2^ > 50%^43, 44^. Subgroup analysis was conducted to explore the sources of the heterogeneity, and sensitivity analysis was performed to examine the effect of excluding each study. Publication bias was examined through visual inspection of a funnel plot, and further evaluated by Begg’s and Egger’s tests^45^ (p < 0.05 indicated a significant publication bias).

## Electronic supplementary material

Supplementary information
