# Supplementary material for: The association between psychological stress and miscarriage: A systematic review and meta-analysis
Source: Sci Rep. 2017 May 11;7:1731. doi: 10.1038/s41598-017-01792-3 (PMC5431920; doi:10.1038/s41598-017-01792-3)
Supplement: Supplementary file 1 — Supplementary information [file 41598_2017_1792_MOESM1_ESM.pdf]

Is maternal psychological stress associated with miscarriage? A systematic review and meta-analysis

Fan Qu [1*], Yan Wu [1*], Yu-Hang Zhu [1*], John Barry [2], Tao Ding [3], Gianluca Baio [3], Ruth Muscat [4], Brenda K. Todd [5], Fang-Fang Wang [1], Paul J Hardiman [2**]

Supplementary Figure S1. Funnel plot of studies meeting inclusion criteria.

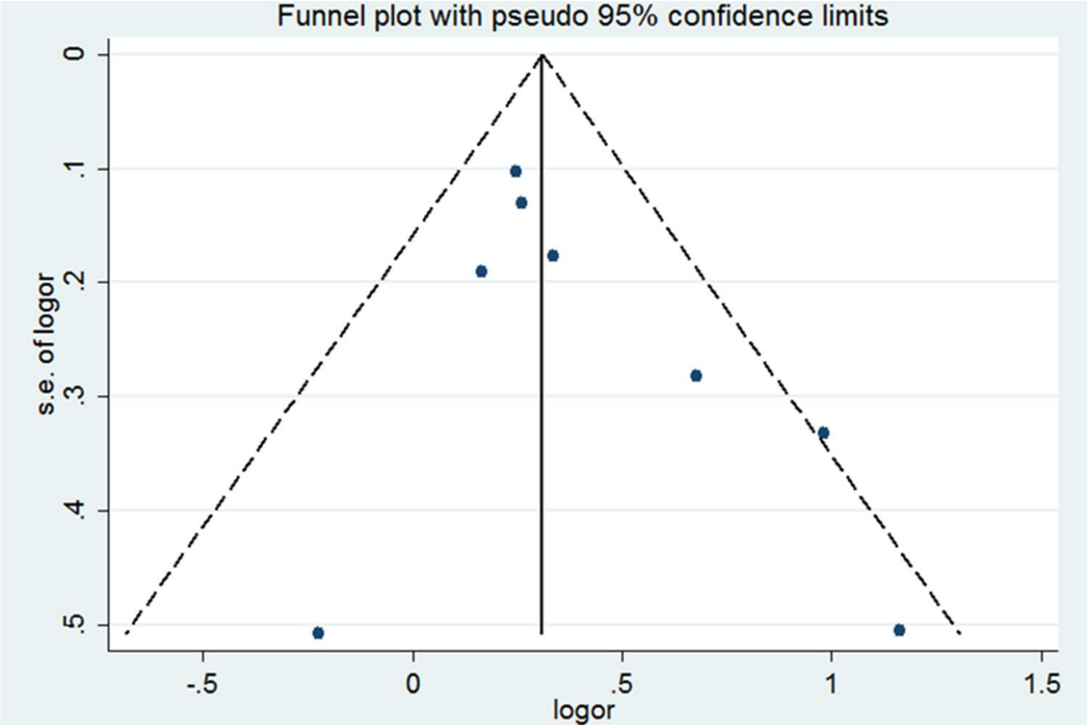

Supplementary Figure S2. Sensitivity analysis of overall meta-analysis

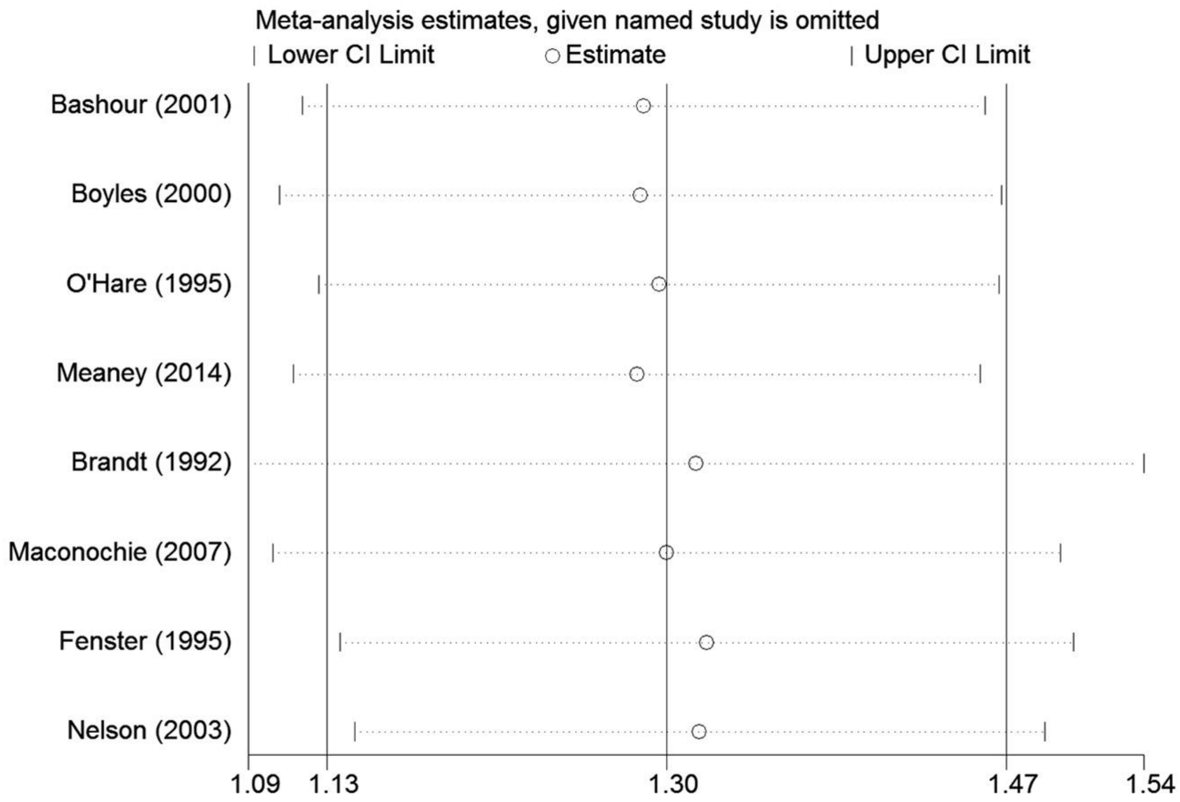

Supplementary Table S1-A: Study Quality assessment using Newcastle-Ottawa scale for cohort studies

Study, Year	Selection				Comparability of cohorts	Outcome			Total score
	Representativeness of exposed cohort	Selection of nonexposed cohort	Ascertainment of exposure	Outcome not present at baseline		Assessment of outcome	Sufficient follow-up duration	Adequate follow-up	
Boyles SH, 2000(Boyles, Ness, Grisso, Markovic, Bromberger and CiFelli, 2000)	*	*	*	*	**	*	*	*	9
Brandt L, 1992(Brandt and Nielsen, 1992)	-	*	*	*	*	-	*	*	6
Fenster L, 1995(Fenster, Schaefer, Mathur, Hiatt, Pieper, Hubbard, Von Behren and Swan, 1995)	*	*	*	*	**	*	*	*	9
Meaney S, 2014(Meaney, Corcoran, Gallagher, Lutomski, Spillane and O'Donoghue, 2014)	*	*	*	*	--	*	*	*	7

Supplementary Table S1-B: Study Quality assessment using Newcastle-Ottawa scale for case-control studies

Study, Year	Selection				Comparability of cases and controls	Exposure			Total Score
	Adequate definition of cases	Representativeness of cases	Selection of controls	Definition of controls		Ascertainment of exposure	Same method of ascertainment for cases and controls	Non-respon se rate	
Bashour H, 2001(Bashour and Abdul Salam, 2001)	*	*	*	-	--	*	*	*	6
Nelson DB, 2003(Nelson, Grisso, Joffe, Brensinger, Shaw and Datner, 2003)	*	*	*	-	**	*	*	*	8
O'Hare T, 1995(O'Hare and Creed, 1995)	*	*	*	-	**	*	*	*	8
Maconochie N, 2007(Maconochie, Doyle, Prior and Simmons, 2007)	*	*	*	-	**	-	*	*	7
